# Investigation of crude oil properties impact on wettability alteration during low salinity water flooding using an improved geochemical model

**DOI:** 10.1038/s41598-022-10506-3

**Published:** 2022-04-22

**Authors:** Maryam Ghorbani, Fariborz Rashidi, Ali Mousavi-Dehghani

**Affiliations:** 1 Petroleum Engineering Department, Amirkabir University of Technology, Tehran, Iran; 2 Chemical Engineering Department, Amirkabir University of Technology, Tehran, Iran; 3 Research Institute of Petroleum Industry, Tehran, Iran

**Keywords:** Energy, Chemical engineering, Geochemistry

## Abstract

In low-salinity water flooding (LSWF), modifying the injected brine composition leads to greater oil recovery from carbonate reservoirs. The processes that control improved recovery during LSWF are not totally clear, which could lead to ambiguities in finding optimum brine composition regarding wettability alteration (WA) toward water wetness. One of the methods to determine WA is bound product sum (BPS) calculation using geochemical tools. In the case of wettability improvement, the BPS value of a crude oil-brine-rock (COBR) system should be at its minimum value. In this study, an improved geochemical model is developed, which includes the effects of oil composition (i.e., acid number, base number, and weight percent of nonhydrocarbon components) and physical properties of oil (i.e., density, viscosity, and solution gas-oil ratio) on COBR interactions. The proposed method generates BPS as a function of temperature, pressure, oil and brine composition, and pH for carbonate rocks. The model applicability was validated using several experimental data sets available in the literature. The results of the improved BPS model were in line with the results of contact angle and zeta potential measurements as the major indices of rock wettability. BPS calculations using the available geochemical tools sometimes failed to predict the correct WA trend since they overlooked the impact of oil properties on COBR interactions. The model predictability was also compared with the results of an available geochemical tool, PHREEQC, and the results demonstrate just how important the effect of oil properties and composition inclusion on wettability determination is. The improved BPS approach could be successfully utilized as an optimization tool to optimize the water composition during LSWF for a given COBR system.

## Introduction

Carbonate reservoirs contain a substantial amount of the world’s oil reserves^1^, but unfortunately, their recoveries are not as high as enough^2^. Laboratory experiments and field trials have shown that enhanced oil recovery from carbonate reservoirs can be obtained by modifying the injected brine composition in a process called low-salinity water flooding (LSWF)^3,4^. LSWF is mostly adopted due to its relative simplicity, low cost^1^, easy implementation, and avoidance complexity of chemical additives such as polymers or surfactants^5^. Typically, the injected brine, in comparison to the formation brine, is of lower salinity and may comprise seawater and/or diluted seawater or low-salinity water with a specific ionic composition^6^.

LSWF influences the wettability and interfacial tension of the COBR system that causes enhanced oil recovery^7^. Due to the existence of some complicated processes in LSWFs, the mechanism by which low salinity water flooding improves oil recovery is not yet well understood, which results in uncertainties in the prediction of optimized low salinity water composition. There have been significant studies to understand the underlying mechanisms controlling LSWF, and several mechanisms have been proposed, including calcite dissolution^8^, the presence of sulfate (and/or dissolution of anhydrite)^4^, and changes in the carbonate mineral surface charge^9^. However, no mechanism has yet been shown to yield improved oil recovery^8^ in all crude oil/brine/rock (COBR) systems; moreover, most fail to explain why published results (and much more unpublished) show no IOR with the dilution of injection brine^3^. The mineral- to pore-scale processes responsible for improved recovery during LSWF^10,11^ remain ambiguous, and there is no definitive method to predict whether a given COBR system will respond to an LSWF or to identify the injection brine composition to maximize oil recovery.

A common tool to identify the effect of a special LSWF is to investigate changes in the zeta potential at the carbonate mineral-brine and oil-brine interface^3,8^. Zeta potential is a measure of the electrical potential at the interface^12^. According to DLVO (Derjaguin, Landau, Verwey, and Overbeek) theory, the electrostatic forces are the summation of van der Waals, electrical double-layer, and structural forces. These forces are generally sensitive to fluid composition, salinity, temperature, and pH^4^. Any change to these parameters will result in a variation in electrostatic forces at the interface. Numerous studies have reported measurements of the zeta potential on both natural and artificial calcite/carbonate surfaces in contact with electrolytes with a range of compositions and total ionic strengths. Decreasing the concentration of divalent ions (such as Mg^2+^ and Ca^2+^) causes an increasingly negative zeta potential^13,14^. The total ionic strength of the brine also affects the magnitude of the zeta potential because it controls the thickness of the diffuse part of the electrical double layer at the mineral-brine interface. Decreasing the total ionic strength decreases the magnitude of the zeta potential in a process often termed double-layer expansion^15^. Despite a large number of published measurements of zeta potential in carbonates^14^, only a few measured data are relevant to carbonate oil reservoirs. Most studies obtain zeta potential data using commercially available laboratory equipment (such as a Zetasizer) that measures electrophoretic mobility^9,16^. These devices have a limited range of operating conditions that are far from those present in real hydrocarbon reservoirs.

The main mechanisms proposed for wettability alteration include fine migration^17^, pH increase leading to interfacial tension reduction^18^, multi-ion exchange, double layer expansion^19^, dissolution of carbonate surface, geochemical processes, and salting in effect. When low salinity water (i.e., of salinity less than 10,000 ppm) is put in contact with the rock surface, the organic components of the rock surface tend to resettle into the aqueous phase. Consequently, the rock tends to disperse water on its surface which results in a water-wet surface^20,21^. Berg et al.^22^ provided direct experimental evidence indicating that multi-ion exchange and/or double layer expansions are potential mechanisms for LSWFs. Wettability alteration processes at pore scale are characterized by the aid of atomic force microscopy, measurements of zeta potential and contact angle, molecular dynamics, etc.^23^. The reduction of cation concentration in the solution causes electrical double layer expansion which results in wettability alteration toward water wetness^24^.

Some simple models merely relate wettability changes to some simple factors, such as the pH^25^ and salinity of injected brine. However, these models will not be able to see the effect of compositional changes in injected brine, which is crucial in optimizing the LSWF. There is another model that includes oil/brine and mineral/brine interactions. Brady and Krumhansl^26^ and Brady et al.^27^ proposed a surface complexation model named BPS in sandstones. This model estimates the sum of the product of oppositely charged surface species (oil/brine and rock/brine surface) concentrations. The concentration of surface aqueous species could be calculated using PHREEQC- a geochemical tool^28^- it considers oil/brine surface reactions and acts them just like other surface reactions such as rock/brine but it does not consider the impact of oil properties, e.g., acid/base number, and its composition on COBR interactions and the consequent wettability state.

In this study, a static geochemical model is developed that considers the effects of oil composition and properties on the COBR interactions. The developed geochemical model in this work covers all mechanisms that affect the wettability alteration in carbonate rocks, including mineral reactions, oil/brine/mineral surface reactions, aqueous phase reactions, and phase equilibrium reactions. Here we are looking for a scientific and less costly solution to detect and quantify the wettability alteration due to geochemical interactions in porous media. Using the BPS concept accompanied with the static geochemical model in this work show that the BPS calculation will give the same results as high-cost contact angle measurements. Furthermore, the range of operating conditions of zeta potential measurements devices to determine wettability alteration is limited^29^ (i.e. up to 80 °C) whereas the model perceives the effect of various pressure and temperature on LSWF since the model parameters are functions of pressure and temperature.

## Model development

The two-phase (oil-brine) model consists of five main reaction groups: phase equilibrium reactions, aqueous species reactions, mineral species reactions, oil surface complexation reactions, and rock surface complexation reactions. The defined reactions of each group are given in Table 1.

**Table 1:** Defined geochemical reactions (inputs to the model).

Phase equilibrium	
$CO_{2\left( o \right)} \Leftrightarrow CO_{{2\left( {aq} \right)}}$	R-1
$N_{2\left( o \right)} \Leftrightarrow N_{{2\left( {aq} \right)}}$	R-2

Aqueous species reactions	
$H_{2} O \Leftrightarrow {\text{H}}^{ + } + {\text{OH}}^{ - }$	R-3
$HCO_{3}^{ - } \Leftrightarrow {\text{H}}^{ + } + {\text{ CO}}_{3}^{2 - }$	R-4
$CaSO_{4} \Leftrightarrow {\text{Ca}}^{2 + } + {\text{ SO}}_{4}^{2 - }$	R-5
$MgSO_{4} \Leftrightarrow {\text{Mg}}^{2 + } + {\text{ SO}}_{4}^{2 - }$	R-6
$NaSO_{4}^{ - } \Leftrightarrow {\text{Na}}^{ + } + {\text{ SO}}_{4}^{2 - }$	R-7
$CaCO_{3} + {\text{ H}}^{ + } \Leftrightarrow {\text{Ca}}^{2 + } + { }HCO_{3}^{ - }$	R-8
$MgCO_{3} + {\text{ H}}^{ + } \Leftrightarrow {\text{Mg}}^{2 + } + { }HCO_{3}^{ - }$	R-9
$CaHCO_{3}^{ + } \Leftrightarrow {\text{Ca}}^{2 + } + { }HCO_{3}^{ - }$	R-10
$MgHCO_{3}^{ + } \Leftrightarrow {\text{Mg}}^{2 + } + { }HCO_{3}^{ - }$	R-11
$NaHCO_{3} \Leftrightarrow {\text{Na}}^{ + } + { }HCO_{3}^{ - }$	R-12

Mineral species reactions	
$Calcite + {\text{ H}}^{ + } \Leftrightarrow {\text{Ca}}^{2 + } + { }HCO_{3}^{ - }$	R-13
$Dolomite + 2{\text{H}}^{ + } \Leftrightarrow {\text{Ca}}^{2 + } + {\text{Mg}}^{2 + } + HCO_{3}^{ - }$	R-14
$Anhydrite + {\text{ H}}^{ + } \Leftrightarrow {\text{Ca}}^{2 + } + { }SO_{4}^{2 - }$	R-15

Surface complexation reactions	
*Rock/brine surface*	
$> CO_{3} H^{0} \Leftrightarrow > CO_{3}^{ - } + { }H^{ + }$	R-16
$> CaOH^{0} + { }H^{ + } \Leftrightarrow > CaOH_{2}^{ + }$	R-17
$> CO_{3} H^{0} + {\text{Ca}}^{2 + } \Leftrightarrow > CO_{3} {\text{Ca}}^{ + } + { }H^{ + }$	R-18
$> CO_{3} H^{0} + {\text{Mg}}^{2 + } \Leftrightarrow > CO_{3} {\text{Mg}}^{ + } + { }H^{ + }$	R-19
$> CaOH_{2}^{ + } + {\text{SO}}_{4}^{2 - } \Leftrightarrow > CaSO_{4}^{ - } + { }H_{2} O$	R-20
$> CaOH^{0} + { }HCO_{3}^{ - } \Leftrightarrow > CaCO_{3}^{ - } + H_{2} O$	R-21

Oil/brine surface	
$- {\text{NH}}^{ + } \Leftrightarrow {\text{H}}^{ + } + { } - {\text{N}}$	R-22
${ } - {\text{COOH}} \Leftrightarrow {\text{H}}^{ + } + { } - COO^{ - }$	R-23
${ } - {\text{COOH}} + {\text{ Ca}}^{2 + } \Leftrightarrow {\text{H}}^{ + } + { } - COOCa^{ + }$	R-24

Details of the geochemical reactions of each group are as follows.

### Phase equilibrium

The equilibrium between the aqueous phase and oil phase is evaluated by equating the fugacity of each component in the two phases. The fugacity of species in the liquid phase is calculated using the Peng-Robinson equation of state assuming an initial composition^30^. The fugacity coefficient yields the fugacity using oil composition and pressure,

1 $$\emptyset_{i,o} = \frac{{f_{i,o} }}{{x_{i} P}}$$

The fugacity of the species in the aqueous phase is calculated using Henry's law^31^:

2 $$\ln H_{i} = \ln H_{i}^{s} + \frac{1}{RT}\mathop \smallint \limits_{{P_{H2O}^{s} }}^{P} \overline{\upsilon }_{i} dP$$

where H_i_ is Henry's law constant for component “i” at pressure P and temperature T. In this equation, $H_{i}^{s}$ is Henry's law constant at water saturation pressure $P_{H2O}^{s}$, is the gas constant, and $\overline{\upsilon }_{i}$. is the partial molar volume of component i in the aqueous phase at T^31^.

3 $$f_{i,aq} = x_{i} H_{i}$$

Thus, the objective function is written as follows:

4 $$R_{i} = \ln K_{i,aq} + \ln \frac{{f_{i,aq} }}{{x_{i,aq} P}} - \ln \frac{{f_{i,o} }}{{x_{i,o} P}}$$

where i refers to common species that can transfer between phases (in this model, N_2_ and CO_2_ are considered soluble components in the oil and water phases).

### Aqueous species reactions

The equilibrium state in the aqueous phase can be calculated by equating the equilibrium constant of the reaction and the activity product of the species:

5 $$R_{\alpha } = Q_{\alpha } - K_{eq,\alpha }$$

where α is the number of aqueous reactions, Q is the activity product of the reaction (aA + bB = cC + dD), in which A and B are reactants, C and D are products, a, b, c, and d are stoichiometric coefficients in the reaction, and ac is the activity coefficient of the aqueous species in the reaction:

6 $$Q = \frac{{ac_{C}^{c} ac_{D}^{d} }}{{ac_{A}^{a} ac_{B}^{b} }}$$

In this model, the activity could alternatively be calculated using a modified Debye–Huckel (also termed the Bdot model, up to an ionic strength of 0.7 mol/kg) or Pitzer model^32^. The equilibrium constant calculation is based on the enthalpy difference, volume difference of reaction, and analytical model which is used in PHREEQC software^28^.

### Mineral reactions

The reaction rate of minerals is calculated through the following equation^33,34^:

7 $$r_{m} = K_{eq,m} \dot{A}_{\beta } \left( {1 - \frac{{Q_{m} }}{{K_{eq,m} }}} \right)$$

where m indicates mineral reactions, Q is the activity product of thmineral equation (the activity of minerals is assumed to be unity) and A_β_ is the specific surface area of the rock. The equilibrium constant of mineral reactions (K_eq,m_) is calculated using the analytical expression used in PHREEQC and using the PHREEQC database^28^.

### Surface complexation reactions

In the surface complexation model, the calcite surface consists of chemically active surface sites that react with ions of the aqueous phase(electrolyte). The strength of these interactions is described by the equilibrium constants. The equilibrium concentration of surface complexes shows the net surface charge and the surface potential^35^. In the rock/brine surface, two main reaction sites are considered > Ca and > CO_3_, where the > sign indicates complexes at the rock surface. Additionally, in the oil/brine contact, two main sites are considered –NH and –COO, where the—sign indicates complexes at the oil surface. The fraction of each surface complexation at each surface site should be calculated using the equality of the equilibrium constant and activity product of surface reactions by considering electrostatic interactions on surfaces^36^. For instance, in the case of the rock/brine surface complexation reaction of R-16 in Table 1, the equilibrium constant is calculated as follows:

8 $$K_{R - 15} = \frac{{g_{{ > CO_{3}^{ - } }} a_{{H^{ + } }} }}{{g_{{ > CO_{3} H^{0} }} }}\chi^{ - 1}$$

where g is the mole fraction of surface complexation species, e.g., for species “i” at the rock or oil surface^36^:

9 $$g_{i} = \frac{{N_{i}^{j} }}{{s_{d}^{j} A_{\beta }^{j} \rho_{j} }} \quad j = oil,rock$$

where N_i_ is the number of moles of complexation species formed at the rock or oil surface. ρ is the bulk rock density or oil density, which is calculated at the desired pressure and temperature employing empirical correlation using the properties of stock tank oil, and s_d_ is the surface site density of rock or oil (i.e., number of surface sites per unit area of the rock surface), which has been estimated to be between 2 and 8 sites/nm^2^ for carbonate rocks^37^. The site density of oil depends on several parameters, such as acid and base number, and A_β_ is the specific surface area of oil or rock. The surface area of oil is proposed to be 0.1 m^2^/g^27^ or 0.11 m^2^/g^19^. The cations of high charge density (i.e., smaller and highly charged) are more hydrated and reluctant to bind to the rock surface and this has a significant role in wettability alteration^38^. The surface area of oil/brine depends on some parameters, such as the acid number/base number ratio and brine composition, because in carbonate rocks, the reactivity of carboxylate groups (–COO) is more important than that of –NH groups since N does not form a stable complex with aqueous species. The residuals of the surface complexation reactions are as follows^39^:

10 $$R_{sc} = \log \frac{{Q_{sc} }}{{K_{sc} }} + \log \chi^{ - 1}$$

The electrostatic interaction term (χ) in the equilibrium constant of surface complexation reactions is:

11 $$\chi = e^{{\frac{{ - zF\psi_{0} }}{RT}}}$$

where F is the Faraday constant, R is the universal gas constant, is the net charge over the surface complexation reaction and ψ_0_ is the surface potential. The surface and zeta potentials at a distance from the surface in the diffuse layer are related by the Gouy-Chapman theory^40^.

The magnitude of the Debye length (a point where ions in the bulk electrolyte solution do not feel electric attraction, the thickness of this diffuse layer is called the Debye length) depends solely on the properties of the solution, not on any properties of the surface, sucas its charge or potential^41^.

It is noticeable that the molality of species is based on the weight of water in the aqueous section of the thin layer between oil and rock. Hence, the portion of water that is involved in the diffuse double layer must be excluded in equilibrium calculations. By defining cation exchange capacity^36^, the mole fraction equation is obtained as follows:

12 $$g_{i} = \frac{{N_{i} }}{\emptyset CEC}$$

For rock/brine surface species, cation exchange capacity, related to the mole fraction of surface species by the above equation, is considered for surface charge calculation as^42^:

13 $$CEC = s_{d} A_{\beta } \rho_{r} \left( {1 - \phi } \right)/\phi$$

where Ø is the porosity of the rock.

For oil/brine surface species, the cation exchange capacity is calculated as:

14 $$CEC = s_{d}^{o} A_{\beta }^{o} \rho_{o} \left( {1 - Sw} \right)$$

where Sw is the saturation of water at the given grid block.

The enrichment factor is useful for modeling the relative enrichme or depletion of equally charged species in the electrostatic layer on a charged surface, which is related to enhanced complexation in a low dielectric permittivity medium^43^.

The acid number and base number strongly affect the oil/brine surface compositions^44^. The base number to acid number ratio (BNANR) is introduced as an enrichment factor in the model. The base number represents the –NH group and the acid number represents the –COOH group. Therefore, the presence of –NH to –COOH groups at the oil/brine surface is related to BNANR.

For the rock/brine surface species, the phase proportion is usually considered to be 0.1 for simulation applications^32^. This means that 0.1 of the moles of minerals participates in the formation of surface complexations.

**Figure 1:**
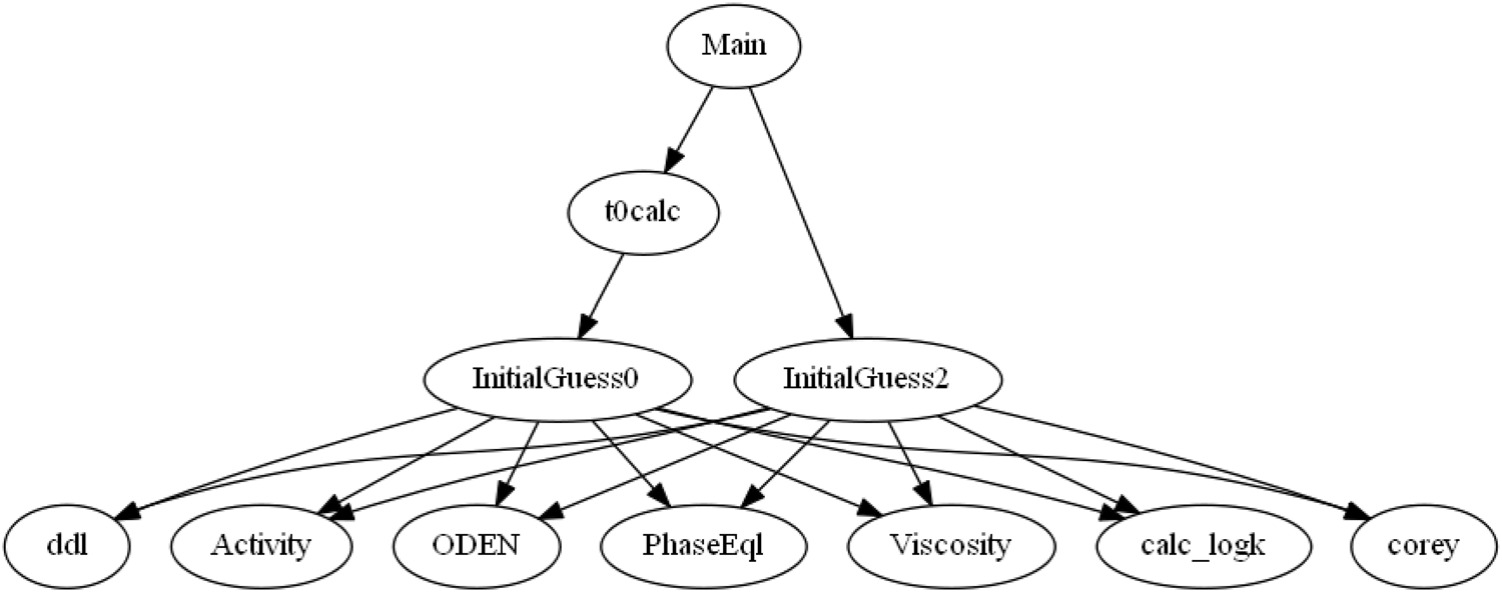
Summary of calculation functions used in the developed model.

The equation set includes the mentioned chemical and thermodynamic equilibria, volume constraint equation (i.e., the total volume of the phases should be equal to the pore volume of the rock), and mass balance equation of the species (i.e., the mass of any species in the pore volume should be equal to the rate of change of its mass). More details on the mass balance and the volume constraint equations are available in the work of Nghiem et al.^45^. The root of the set of nonlinear algebraic equations representing the whole geochemical system at equilibrium should be solved. The system of equations is solved using a trust-region-reflective algorithm and gives the equilibrium concentrations of species. A summary of reactive transport code functions is illustrated in Fig. 1. The application of the functions is presented as follows: **Main** is for the first guess calculation, advancing time steps, and plotting the results, **t0Calc** is for first guess calculation using initial values in **Initialguess0** function, **Initialguess2** is for the calculation of iterative time step values, **Activity** calculates activity using Bdot or Pitzer model, **ODEN** calculates oil density using oil API and dissolved gas-oil ratio, **Viscosity** calculates the viscosity of brine using salinity (or TDS) and oil at the desired pressure and temperature using physical properties of oil (i.e., oil density, dissolved gas-oil ratio, and bubble point pressure), **PhaseEql** is a function of phase equilibrium calculations using Peng-Robinson and Henrys’ constant calculations, **corey** returns the relative permeability of oil and water and capillary pressure using Brooks-Corey’s equilibrium model, **calc_logk** calculates the equilibrium constants of reactions through the analytical model of PHREEQC, ddl calculates the portion of water that exists in the diffuse double layer and must be excluded in the concentration calculations based on the mass of water.

**Figure 2:**
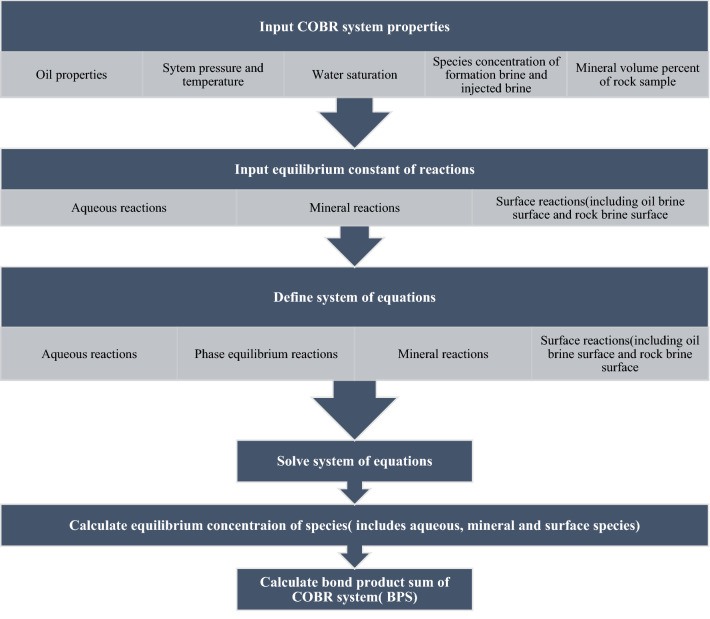
Flowchart of the developed model for wettability determination using BPS calculation.

The wettability of the COBR system is determined using a surface complexation model that is considered an improved BPS approach. The flowchart of the developed model for wettability determination using BPS calculation is presented in Fig. 2. In the following section, the BPS calculation of a given COBR system is discussed.

## Surface complexation model

Surface complexation modeling presumes an electrical double layer at each interface and the existence of charged surface species whose concentrations depend upon the chemical makeup of water, oil, and mineral surfaces^33,46^.

## Oil–brine surface charge

The diffuse layer model is a simple model for the electric double layer and envisions a surface layer with a pH-dependent surface charge and a Gouy-Chapman diffuse layer of oppositely charged ions; see, e.g., Dzombak and Morel^47^, with the sorbed ions residing on the surface layer.

Measured zeta potentials of the oil-brine interface could be represented through the sum of carboxylic acids and nitrogen bases^26^. In the oil/brine surface reactions, –COOH and –NH represent dangling carboxylate and nitrogen base groups present at the oil–water interface. At pH > 4.9, any rise in the ionic strength will increase the number of deprotonated carboxylate groups and interfacial negative charges, while at pH < 4.9, any rise in the ionic strength will increase the number of protonated nitrogen bases and positive surface charges. The absolute value of oil-brine interface zeta potential is decreased by increasing the ionic strength of the solution^27^. Relative numbers of acid and base groups in the model may be changed to consider oils with different acid and base numbers. These parameters, along with many others, will affect surface complex concentrations involving reactions of a COBR system that controls the wettability of rock. LSWF is successful only if the zeta potential changes at the mineral-brine interface become more negative (i.e. decrease ionic strength) when the oil-brine interface has a negative zeta potential in the formation brine^48^.

A quantitative measure of electrostatic attraction is termed BPS^27,32^. This electrostatic attraction causes oppositely charged species to attract each other, leading to an oil-wet surface. BPS is a reliable index that indicates the measure of the electrostatic bond between oppositely charged surface complexes, which could also be an indicator of wettability or tendency of oil to stick on the rock surface; therefore, BPS contains electrostatic charge and surface potential and interaction of different species together.

The lower limit of BPS is “zero”, meaning there is no electrostatic bound between surface species. Therefore, low values of BPS indicate a water-wet system of COBR. In this model, BPS is calculated as follows (absolute unit is mol^2^ and density unit is (μmol/m^2^)^2^):

15 $$\begin{aligned} {\text{BPS}} = & \left[ { - {\text{NH}} + } \right]{\text{ }}*\left( {\left[ { > {\text{CO}}_{{\text{3}}} ^{ - } } \right]{\text{ }} + \left[ { > {\text{CaSO}}_{{\text{4}}} ^{ - } } \right]{\text{ }} + \left[ { > {\text{CaCO}}_{{\text{3}}} ^{ - } } \right]} \right){\text{ }} \\ & \quad + \left[ { - {\text{COO}}^{ - } } \right]{\text{ }}*\left( {\left[ { > {\text{CaOH}}_{{\text{2}}} ^{ + } } \right]{\text{ }} + \left[ { > {\text{CO}}_{{\text{3}}} {\text{Ca}}^{ + } } \right]{\text{ }} + \left[ { > {\text{CO}}_{{\text{3}}} {\text{Mg}}^{ + } } \right]} \right) \\ & \quad + \left[ { - {\text{COOCa}}^{ + } } \right]{\text{ }}*(\left[ { > {\text{CO}}_{{\text{3}}} ^{ - } } \right]{\text{ }} + \left[ { > {\text{CaSO}}_{{\text{4}}} ^{ - } } \right]{\text{ }} + [ > {\text{CaCO}}_{{\text{3}}} ^{ - } ]) \\ \end{aligned}$$

**Figure 3:**
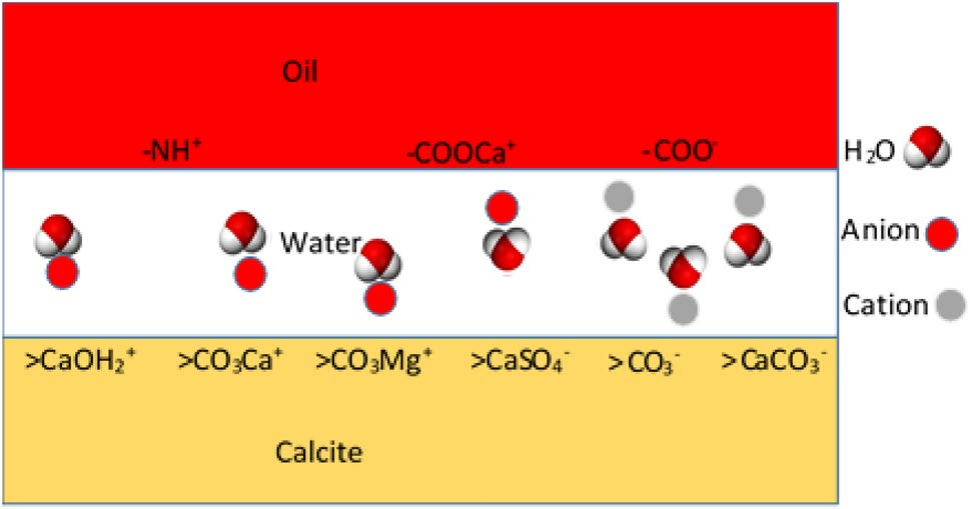
Schematic of the electrostatic interaction between ions in an oil-brine-calcite system.

BPS values should decrease for wettability improvement (i.e., more water-wet state), based on Brady et al.^26^. A schematic of the electrostatic interaction between ions in a COBR system is shown in Fig. 3.

The system of nonlinear equations is solved using a trust-region-reflective algorithm, so the equilibrium state of reactions is determined. First, it should be clarified that the wettability alteration trend of a given COBR system is predicted correctly by employing the developed model, so the model validation is performed using several experimental data sets in the literature that are discussed in the following section. Next, method validation is performed through a comparison between the predictability of the model and an available geochemical tool, PHREEQC^28^.

## Model validation

Two experimental studies were used to validate the results from the model.

### Effect of injected brine composition on calculated BPS

The contact angle is a measure of wettability, which is the angle of the fluid–fluid interface. If the contact angle of one fluid is smaller than 90°, the solid is said to be preferentially wet by this fluid^49^. Alshakhs and Kovscek^18^ conducted zeta potential measurements on calcite rock (different pH values of injected brine) to calculate contact angle employing DLVO theory using disjoining pressure calculations.

The petrophysical properties of the core samples used for core flooding are shown in Table 2, and the properties of the crude oil are shown in Table 3. The ionic composition of different injected brines is shown in Table 4. The temperature of the experiments was 60 °C.

**Table 2:** Petrophysical properties of core samples.

Rock type	Sample	Porosity (%)	Permeability^14^	S_wi_	Size
Carbonate	SU-1	20.7	554	11	1.5ʺ dim × 2ʺ

**Table 3:** Fluid properties of crude oil.

Oil type	API°	Viscosity (at 80 °C), cP	Acid#, mg KOH/g	Base#, mg KOH/g
Crude oil	38.2	2.4	1.15	1.25

**Table 4:** Injected brine compositions and compositions of ions are in kppm units.

Ions	1	2	3	4
Sodium	17.2	10.2	8.1	10.2
Magnesium	2.1	3.5	3.5	0
Sulfate	4.3	8.9	0	8.9
Chloride	31	22.5	22.5	22.5
TDS	54.6	45.1	34.3	31.5
Ionic strength, mole/L	1.074	1.008	0.775	0.582
Molarity, M	0.82	.59	0.5	0.44

At pH = 8.5, the measured and calculated contact angle is shown in Table 5.

**Table 5:** Reported contact angle by Alshakhs and Kovscek^18^.

pH = 8.5, I = 0.001 Injection scenario	Contact angle
1	33.97
2	29.21
3	29.85
4	33.72

Sequence of contact angle values of injection scenarios at this pH is: $\theta_{1} > \theta_{4} > \theta_{3} > \theta_{2}$.

The estimated BPS[mol^2^] values using the model are shown in Table 6.

**Table 6:** Calculated BPS using the developed model.

pH = 8.5, I = 0.001 Injection scenario	BPS(μmol^2^)
1	3.56E+04
2	2.68E+04
3	2.72E+04
4	3.19E+04

**Figure 4:**
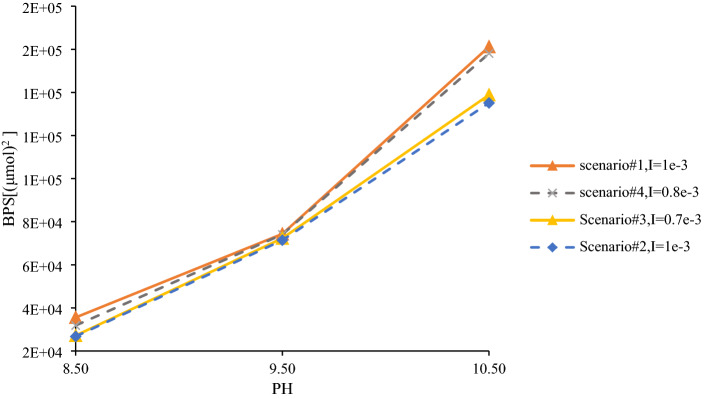
Calculated BPS of brines injected using compositions of Alshakhs and Kovscek’s work^18^.

The sequence of BPS values of injection scenarios at this pH is $BPS_{1} > BPS_{4} > BPS_{3} > BPS_{2}$, which implies that BPS correctly models the wettability alteration during injection of different composition brines and indicates that the presence of MgSO_4_^2−^ in the composition of injected brine has a positive effect in altering wetness toward water wetting, while SO_4_^2−^ has very little effect. In the case of injection scenario 4 relative to 1, it is clear that negatively charged SO_4_^2−^ attracts the positively charged calcite rock surface; therefore, there are more water-wet sites for the negatively charged oil surface. Injection scenario 3 causes calcite dissolution due to the higher activity of Mg^2+^ relative to rock Ca^2+^ ions, and consequently, injection scenario 2 results in more wettability improvement due to the simultaneous effect of SO_4_^2−^ attraction and dissolution of calcite. It seems that the improved model captures the mentioned effects concurrently since there is a satisfactory agreement between the model results and the experimental results. The calculated BPS values using the model are presented in Fig. 4. By decreasing PH of the solution, concentration of H + ions increases, causing the reaction between divalent ions of formation brine (Ca2 + , Mg2 +) with the solution to decrease^50^. This results in concentration decreasing of carboxylate groups (–COO^−^) on oil surface which leads to water-wet state. Consequently, by consideration of positively charged calcite surface, the sum of oppositely charged complexes at the oil-brine-rock interfaces decreases that means BPS reduction.

### Effect of temperature on calculated BPS

The developed model was also validated using the results of Mahani et al.^19^ experiments. They conducted zeta potential and contact angle measurements on several carbonate rock samples at different temperatures. The injected brine was 25 times diluted seawater, and its composition is shown in Table 7.

**Table 7:** Ionic composition of injected brine, as reported by Mahani et al.^19^.

Na^+^(mg/l)	536
K^+^(mg/l)	19
Mg^2+^(mg/l)	65
Ca^2+^(mg/l)	20
Sr^2+^(mg/l)	1
Cl^-^(mg/l)	967
SO_4_^2−^(mg/l)	135
HCO_3−_ (mg/l)	7
Total dissolve solids(ppm)	1751
Ionic strength(mol/l)	0.035
pH	7.5

The calcite site density and specific surface area are considered 2 sites/nm^2^ and 0.11 m^2^/g, respectively. The physical and chemical properties of crude oil A are shown in Table 8.

**Table 8:** Crude oil properties, as reported by Mahani et al.^19^.

Oil sample	Acid number (mg KOH/g)	Base number (mg KOH/g)	Asphaltene (g/100 mL)	Density (g/cm^3^) at 20 °C	Viscosity (cP) at 20 °C
Crude oil A	0.5	1	0.244	0.865	20.7

It was reported that in limestone A (only calcite rock), increasing the temperature did not show any wettability improvement, whereas dolomite (99% dolomite + 1% quartz) showed wettability improvement by injecting 25 times diluted seawater (25dSW).

The calculated BPS values using the developed code are presented in Table 9.

**Table 9:** The calculated BPS of rock samples of Mahani et al.^19^ at different temperatures.

Rock sample	BPS(μmol^2^)	Temperature(°C)
Limestone A(only calcite rock)	2.51E+06	25
	2.51E+06	80
Dolomite(mainly dolomite + 1%quartz)	9.05E+05	25
	6.34E−02	80

**Figure 5:**
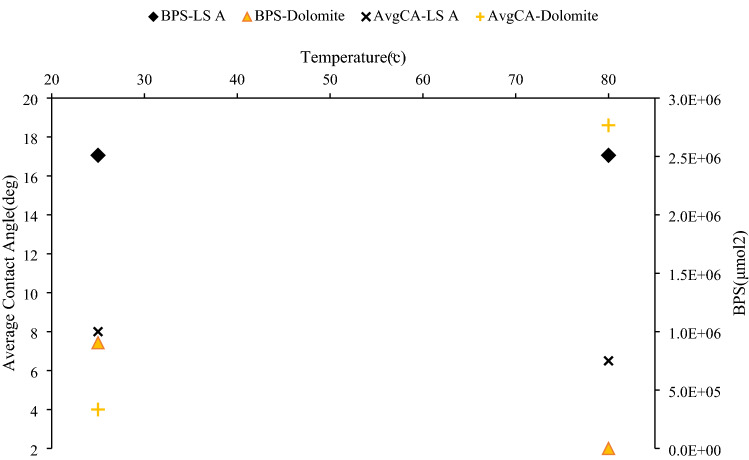
Calculated BPS of limestone A and dolomite (BPS-LS A and BPS-dolomite) and average contact angle change of the rocks (AvgCA-LS A and AvgCA-dolomite) reported by Mahani et al.^19^.

As shown in Table 9, in the case of dolomite rock type, temperature rise causes improvement in wettability through water wetting, whereas in the case of calcite rock, increasing the temperature has a negligible effect on wettability. The calculated BPS values of Mahani et al.’s work^19^ and the average contact angle changes of the two rock samples (limestone A and dolomite), reported in his work, are illustrated in Fig. 5. The contact angle change is the difference between the contact angle of an oil droplet at the time of equilibration with formation water and the end of exposure to 25dSW.

Different mechanisms for wettability alteration have been proposed in the literature^4,36,51^ that include altering the surface charge through adsorption of Ca^2+^ and SO_4_^2−^ on the chalk surface and Ca^2+^ substitution by Mg^2+^ due to increasing ion reactivity at higher temperatures. By injecting brines containing SO_4_^2−^, it is assumed that this ion will be adsorbed on a positively charged chalk surface. Thus, the bond between the negatively charged oil surface and the positively charged surface will break down. This effect represents a correlation between oil recovery and temperature in spontaneous imbibition tests, through intensifying this effect by increasing temperature^52^. It is proposed that at high temperatures, the reactivity of these ions increases, and Ca^2+^ on the rock surface will be substituted by Mg^2+^.

These observations are thoroughly included in modeled reactions through activity coefficient calculations, equilibrium constant databases, and molar volume changes in the reactions, which are functions of pressure and temperature. However, to the best of the authors’ knowledge, the effect of increasing pressure on wettability alteration has not been presented in the literature before use in model validation. The effect of pressure on the reaction is incorporated through the molar volume change of the reaction that affects the equilibrium constant calculation, although the fugacity of the phases is affected by pressure. Therefore, the pressure effect is also incorporated into the phase equilibrium calculations. This finding confirms the usefulness of the new model as an optimization tool that optimizes injected brine composition by considering the effect of various parameters, such as pressure, temperature, and oil properties. On the equilibrium concentration of all species.

Measured zeta potentials, due to the evaporation of brine at high temperatures, are much more stable at 25 °C. Hence, zeta potentials at 25 °C are chosen to examine the surface potential dependencies of oil and rock surface reactivity at higher temperatures, assuming that zeta potentials tend to decrease uniformly with an increase in temperature^33^. There are a few measured zeta potential data in the range of oil reservoir temperatures (higher than 100 °C), and the existence of a model could be of assistance to optimize brine compositions and be employed to inject low salinity water flooding under reservoir conditions.

### Comparison between predictability of the model and PHREEQC

Korrani^53^ tested the BPS approach predictability for several modified salinity waterfloods, such as the experimental work by Yousef et al.^51^. Korrani^53^ used PHREEQC to calculate BPS of different dilutions of injected water. He reported that BPS failed to predict the correct trend for Yousef et al.^51^ experiments. The present study conducted BPS computation assuming that the base number of Yousef et al.^51^ crude oil is 1 mg KOH/g of oil, which was not reported by the authors. The temperature of the experiments was 100 °C. All other properties regarding core, oil, and brine were the same as those reported in their work.

**Figure 6:**
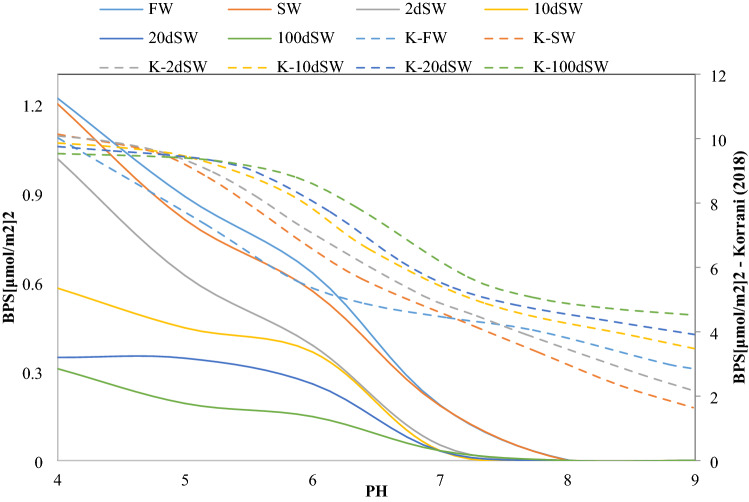
Calculated BPS for brines used in **Yousef et al**.^51^’s core flood using the developed model compared to BPS values extracted from **Korrani**^53^, axes on right and dashed line on legend are data extracted from Korrani^53^’s work, solid lines are data calculated using the developed model.

For the examined dilutions of seawater presented in Yousef et al.^51^, BPS was calculated in density units, and the results are shown in Fig. 6. The reported contact angle by Yousef et al.^51^ is shown in Table 10.

**Table 10:** Reported contact angle by Yousef et al.^51^.

Brine	Contact angle
Connate water	90
Seawater	90
Twice diluted seawater	80.9
10 times diluted seawater	69.2
20 times diluted seawater	63
100 times diluted seawater	62.2

It is clear that since BPS has a little positive response (i.e., wettability improvement or BPS reduction) compared to formation brine, injecting seawater has little effect on wettability. However, two- and tenfold dilutions of seawater (2dsw and 10dsw, respectively) have a significant effect on the BPS trend since at a constant pH, seawater causes smaller BPS values than formation brine. It is also reported that 20 times and 100 times dilutions of seawater (20dsw and 100dsw, respectively) have a lesser effect on the BPS trend in comparison to the previous dilutions (10dSW and 20dSW, respectively). These observations correspond to the experimental work by Yousef et al.^51^.

Dilution of seawater leads to ionic strength reduction, which is directly related to the ionic activity coefficient. Thus, the interaction between ions decreases, which causes interfacial tension reduction and subsequent wettability improvement. As already mentioned, in the existing geochemical tools, such as PHREEQC^28^, the effect of oil phase properties and compositions is excluded, whereas, in the developed model, it is included in the equilibrium calculations. This comparison emphasizes the method validation and indicates that the presented model could effectively determine wettability alteration relative to the existing geochemical tools.

## Conclusions

This study presents an improved BPS approach for determining wettability alterations of carbonate rocks by calculating the BPS values of a given COBR system. The effect of oil properties (i.e., density, viscosity, and solution gas-oil ratio) and composition (i.e., acid number, base number, and weight percent of nonhydrocarbon components) is included in COBR interactions. Reactions between phases and all aqueous, mineral, oil, and surface species are coupled in the developed geochemical model, and the system of equations is solved to give equilibrium concentrations of species. BPS values are calculated using the computed equilibrium concentration of species for a given COBR system. The results of the developed model are validated using several published experimental works. The following conclusions were drawn from the study:

1. The developed program considers the effect of oil composition and its physical properties on COBR interactions and wettability.
2. The proposed method conducted phase equilibrium calculations, while it considered the effect of soluble components of each phase on the composition and thickness of the diffused double layer (since the portion of water that is involved in the diffuse double layer must be excluded in equilibrium calculations). Subsequently, it generates BPS as a function of temperature, pressure, oil and brine composition, and pH for carbonate rocks.
3. The developed model is validated using experimental data in the literature; contact angle alteration in the two-phase oil-brine system of different brine compositions showed the same trend as experimental results using zeta potential measurements.
4. Contact angle alteration in the two-phase oil-brine system at different temperatures showed the same trend as the experimental results using zeta potential and contact angle measurements. Therefore, the developed model could successfully determine wettability alteration by considering the temperature effect on the equilibration of phases and species.
5. The results of BPS calculations using PHREEQC are compared to the results of the developed program for the oil-brine-rock system of experimental work by Yousef et al.^24^. This result supports the idea that oil phase properties and composition inclusion are of great importance in phase and species equilibrium calculations.
6. The results suggest that the improved BPS approach could be successfully utilized as an optimization tool to optimize the water composition during LSWF since BPS (which indicates the wettability) is calculated as a function of pressure, temperature, oil and brine compositions, and pH for a given COBR system.

## Data Availability

The data will be available upon request.

## References

[1] Jha NK (2021). Interaction of low salinity surfactant nanofluids with carbonate surfaces and molecular level dynamics at fluid-fluid interface at ScCO2 loading. J. Colloid Interface Sci..

[2] Song J (2020). Evaluating physicochemical properties of crude oil as indicators of low-salinity–induced wettability alteration in carbonate minerals. Sci. Rep..

[3] Austad T, Shariatpanahi S, Strand S, Black C, Webb K (2012). Conditions for a low-salinity enhanced oil recovery (EOR) effect in carbonate oil reservoirs. Energy Fuels.

[4] Moghadasi R, Rostami A, Tatar A, Hemmati-Sarapardeh A (2019). An experimental study of Nanosilica application in reducing calcium sulfate scale at high temperatures during high and low salinity water injection. J. Petrol. Sci. Eng..

[5] Moeini F, Hemmati-Sarapardeh A, Ghazanfari M-H, Masihi M, Ayatollahi S (2014). Toward mechanistic understanding of heavy crude oil/brine interfacial tension: The roles of salinity, temperature and pressure. Fluid Phase Equilib..

[6] Divandari H, Hemmati-Sarapardeh A, Schaffie M, Ranjbar M (2020). Integrating functionalized magnetite nanoparticles with low salinity water and surfactant solution: Interfacial tension study. Fuel.

[7] Honarvar B, Rahimi A, Safari M, Khajehahmadi S, Karimi M (2020). Smart water effects on a crude oil-brine-carbonate rock (CBR) system: Further suggestions on mechanisms and conditions. J. Mol. Liq..

[8] Hiorth A, Cathles L, Madland M (2010). The impact of pore water chemistry on carbonate surface charge and oil wettability. Transp. Porous Media.

[9] Jackson MD, Al-Mahrouqi D, Vinogradov J (2016). Zeta potential in oil-water-carbonate systems and its impact on oil recovery during controlled salinity water-flooding. Sci. Rep..

[10] Khishvand M, Alizadeh A, Kohshour IO, Piri M, Prasad R (2017). In situ characterization of wettability alteration and displacement mechanisms governing recovery enhancement due to low-salinity waterflooding. Water Resour. Res..

[11] Aziz R (2019). Novel insights into pore-scale dynamics of wettability alteration during low salinity waterflooding. Sci. Rep..

[12] Zhang P, Tweheyo MT, Austad T (2007). Wettability alteration and improved oil recovery by spontaneous imbibition of seawater into chalk: Impact of the potential determining ions Ca2+, Mg2+, and SO42−. Colloids Surf. A.

[13] Al Mahrouqi D, Vinogradov J, Jackson MD (2017). Zeta potential of artificial and natural calcite in aqueous solution. Adv. Colloid Interface Sci..

[14] Alroudhan A, Vinogradov J, Jackson M (2016). Zeta potential of intact natural limestone: Impact of potential-determining ions Ca, Mg and SO4. Colloids Surf. A.

[15] Hunter RJ (2013). Zeta Potential in Colloid Science: Principles and Applications.

[16] Van Cappellen P, Charlet L, Stumm W, Wersin P (1993). A surface complexation model of the carbonate mineral-aqueous solution interface. Geochim. Cosmochim. Acta.

[17] Tang G-Q, Morrow NR (1999). Influence of brine composition and fines migration on crude oil/brine/rock interactions and oil recovery. J. Pet. Sci. Eng..

[18] Alshakhs MJ, Kovscek AR (2016). Understanding the role of brine ionic composition on oil recovery by assessment of wettability from colloidal forces. Adv. Colloid Interface Sci..

[19] Mahani H (2017). Insights into the impact of temperature on the wettability alteration by low salinity in carbonate rocks. Energy Fuels.

[20] Rahimi A, Honarvar B, Safari M (2020). The role of salinity and aging time on carbonate reservoir in low salinity seawater and smart seawater flooding. J. Pet. Sci. Eng..

[21] Yisong L, Gholami R, Safari M, Rahimi A, Siaw Khur W (2022). On surface interactions of environmental friendly surfactant/oil/rock/low salinity system: IFT, wettability, and foamability. J. Pet. Sci. Eng..

[22] Berg S, Cense A, Jansen E, Bakker K (2010). Direct experimental evidence of wettability modification by low salinity. Petrophys. SPWLA J. Form. Eval. Reserv. Descr..

[23] Chen Y (2020). Geochemical controls on wettability alteration at pore-scale during low salinity water flooding in sandstone using X-ray micro computed tomography. Fuel.

[24] Safari M, Rahimi A, Gholami R, Permana A, Siaw Khur W (2022). Underlying mechanisms of shale wettability alteration by low salinity water injection (LSWI). J. Dispers. Sci. Technol..

[25] Hilner E (2015). The effect of ionic strength on oil adhesion in sandstone – the search for the low salinity mechanism. Sci. Rep..

[26] Brady PV, Krumhansl JL (2012). A surface complexation model of oil–brine–sandstone interfaces at 100 C: Low salinity waterflooding. J. Pet. Sci. Eng..

[27] Brady PV, Thyne G (2016). Functional wettability in carbonate reservoirs. Energy Fuels.

[28] Parkhurst DL, Appelo C (1999). User's guide to PHREEQC (Version 2): A computer program for speciation, batch-reaction, one-dimensional transport, and inverse geochemical calculations. Water-Resour. Investig. Rep..

[29] Delgado AV, González-Caballero F, Hunter RJ, Koopal LK, Lyklema J (2007). Measurement and interpretation of electrokinetic phenomena. J. Colloid Interface Sci..

[30] Danesh A (1998). PVT and Phase Behaviour of Petroleum Reservoir Fluids.

[31] Harvey AH (1996). Semiempirical correlation for Henry's constants over large temperature ranges. AIChE J..

[32] Zhang G, Zheng Z, Wan J (2005). Modeling reactive geochemical transport of concentrated aqueous solutions. Water Resour. Res..

[33] Xie Q (2017). The low salinity effect at high temperatures. Fuel.

[34] Zhang Y, Hu B, Teng Y, Tu K, Zhu C (2019). A library of BASIC scripts of reaction rates for geochemical modeling using phreeqc. Comput. Geosci..

[35] Vinogradov J (2022). Predictive surface complexation model of the calcite-aqueous solution interface: The impact of high concentration and complex composition of brines. J. Colloid Interface Sci..

[36] Pokrovsky O, Schott J (2002). Surface chemistry and dissolution kinetics of divalent metal carbonates. Environ. Sci. Technol..

[37] Puntervold T (2018). Role of kaolinite clay minerals in enhanced oil recovery by low salinity water injection. Energy Fuels.

[38] Jha NK (2019). Wettability alteration of quartz surface by low-salinity surfactant nanofluids at high-pressure and high-temperature conditions. Energy Fuels.

[39] McNeece CJ, Hesse MA (2016). Reactive transport of aqueous protons in porous media. Adv. Water Resour..

[40] Adamson AW, Gast AP (1967). Physical Chemistry of Surfaces.

[41] Israelachvili JN (2011). Intermolecular and Surface Forces.

[42] Revil A (2017). Transport of water and ions in partially water-saturated porous media. Part 2. Filtration effects. Adv. Water Resour..

[43] Appelo C, Van Loon L, Wersin P (2010). Multicomponent diffusion of a suite of tracers (HTO, Cl, Br, I, Na, Sr, Cs) in a single sample of Opalinus Clay. Geochim. Cosmochim. Acta.

[44] Sari A, Chen Y, Xie Q, Saeedi A (2019). Low salinity water flooding in high acidic oil reservoirs: Impact of pH on wettability of carbonate reservoirs. J. Mol. Liq..

[45] Nghiem, L., Sammon, P., Grabenstetter, J. & Ohkuma, H. In *SPE/DOE Symposium on Improved Oil Recovery.*

[46] Lutzenkirchen J (2006). Surface Complexation Modelling.

[47] Dzombak DA, Morel FM (1991). Surface Complexation Modeling: Hydrous Ferric Oxide.

[48] Jackson MD, Al-Mahrouqi D, Vinogradov J (2016). Zeta potential in oil-water-carbonate systems and its impact on oil recovery during controlled salinity water-flooding. Sci. Rep..

[49] Zhao J (2018). Lattice Boltzmann simulation of liquid flow in nanoporous media. Int. J. Heat Mass Transf..

[50] Honarvar B, Rahimi A, Safari M, Rezaee S, Karimi M (2019). Favorable attributes of low salinity water aided alkaline on crude oil-brine-carbonate rock system. Colloids Surf. A.

[51] Yousef AA, Al-Saleh S, Al-Kaabi A, Al-Jawfi M (2011). Laboratory investigation of the impact of injection-water salinity and ionic content on oil recovery from carbonate reservoirs. SPE Reserv. Eval. Eng..

[52] Pokrovsky OS, Schott J, Thomas F (1999). Dolomite surface speciation and reactivity in aquatic systems. Geochim. Cosmochim. Acta.

[53] Korrani AK, Jerauld GR (2019). Modeling wettability change in sandstones and carbonates using a surface-complexation-based method. J. Pet. Sci. Eng..

